# Usefulness of International Normalized Ratio to Albumin Ratio for Evaluation of Mortality in Hepatitis B Virus-Associated Decompensated Cirrhosis

**DOI:** 10.1155/2021/6664574

**Published:** 2021-05-11

**Authors:** Ming Cai, Zhong Han, Xia He, JinFei Zhang

**Affiliations:** Department of Clinical Laboratory, Shengzhou People's Hospital, Shengzhou Branch of the First Affiliated Hospital of Zhejiang University, Shengzhou 312400, China

## Abstract

**Background:**

We sought to determine the prognostic value of prothrombin time-international normalized ratio to albumin ratio (PTAR) in patients with hepatitis B virus-associated decompensated cirrhosis (HBV-DeCi).

**Methods:**

The study enrolled 166 HBV-DeCi patients. Multivariate regression analysis was performed to identify predictors associated with mortality.

**Results:**

Among the 166 HBV-DeCi patients, 27 (16.3%) had died by 30 days after admission. PTAR was markedly increased in nonsurvivors compared with survivors, and had a significant positive correlation with disease severity. Multivariate analysis identified PTAR as an effective independent predictor for mortality in HBV-DeCi patients.

**Conclusions:**

High PTAR was associated with poor outcomes and can act as a novel prognostic predictor for mortality in HBV-DeCi patients.

## 1. Introduction

Hepatitis B virus (HBV) infection remains a major public health problem with significant morbidity and mortality rates [1]. In China, HBV infection is the predominant cause of liver cirrhosis, and approximately 2–5% of HBV-infected patients with compensated cirrhosis develop decompensated cirrhosis (DeCi) each year [2]. A previous study reported that the 5-year survival rate is only 14%–35% once decompensation has occurred [3]. Liver transplantation is a reliable and effective intervention for patients suffering from this condition. However, the lack of liver sources and serious posttransplantation complications have limited its application. Therefore, an accurate, convenient, and noninvasive prognostic biomarker is needed to identify the mortality risk for HBV-DeCi patients, with a view to stratifying and improving their clinical management and survival.

The prothrombin time-international normalized ratio (PT-INR) to albumin ratio (PTAR) was initially reported by Haruki et al. for evaluation of the liver functional reserve in patients with hepatocellular carcinoma (HCC) after liver resection [4]. They demonstrated that PTAR could reflect liver function and act as a novel predictive factor for adverse outcomes in these patients. INR and albumin both reflect the synthetic function of the liver, and common abnormalities in these two indicators often lead to a poor prognosis in cirrhotic patients [5]. PTAR was also identified as a novel independent predictor of adverse outcomes in critically ill patients with cirrhosis [6]. However, few studies have evaluated the role of PTAR in HBV-DeCi patients. Thus, we performed a retrospective study to determine the prognostic role of PTAR in these patients.

## 2. Materials and Methods

### 2.1. Patients

A total of 205 HBV-DeCi patients treated in our hospital between May 2014 and May 2016 were retrospectively assessed. All patients had shown positivity for HBV surface antigens for >6 months. DeCi was defined by the development of hepatic encephalopathy, ascites, gastrointestinal bleeding, hepatorenal syndrome, or any combination of these [7]. The exclusion criteria were as follows: age < 18 years or >75 years; coinfection with other hepatitis virus or HIV, or concurrent autoimmune or other liver diseases; HCC or malignancy; hematological diseases; incomplete data at admission; and treatment with immunomodulatory therapy within 3 months before admission. All patients were followed up for 30 days to assess the 30-day survival status. The study was approved by the ethical committee of Shengzhou People's Hospital.

### 2.2. Data Collection

For each patient, demographic information and laboratory data were collected on admission. Laboratory parameters, including alanine aminotransferase, aspartate aminotransferase, total protein, albumin, creatinine, total bilirubin, INR, platelet count, hemoglobin, and blood urea nitrogen, were collected. Biochemical analyses were conducted using a Hitachi 7600 Clinical Analyzer (Hitachi, Tokyo, Japan), Sysmex CA-7000 System (Sysmex, Kobe, Japan), and Sysmex XE-2100 Auto-Analyzer (Sysmex, Kobe, Japan) using standard methods. PTAR was calculated as INR divided by albumin (g/dL). Hepatic disease severity was evaluated by the Model for End-Stage Liver Disease (MELD) score as previously described [8].

### 2.3. Statistical Analysis

Statistical analyses were carried out using SPSS version 17.0 or MedCalc version 12.7 software. Two-sided *P* < 0.05 was considered to indicate statistical significance. Continuous data were expressed as median with interquartile range and analyzed using the Mann–Whitney *U* test. Categorical data were expressed as number and analyzed using Fisher's exact test. The correlation between PTAR and MELD score was determined by Spearman's rank correlation analysis. Independent predictors for mortality were identified by univariate and multivariate analyses. The areas under the curve (AUCs) were measured and compared to evaluate the discrimination abilities of PTAR and MELD score.

## 3. Results

### 3.1. Study Population

**Figure 1:**
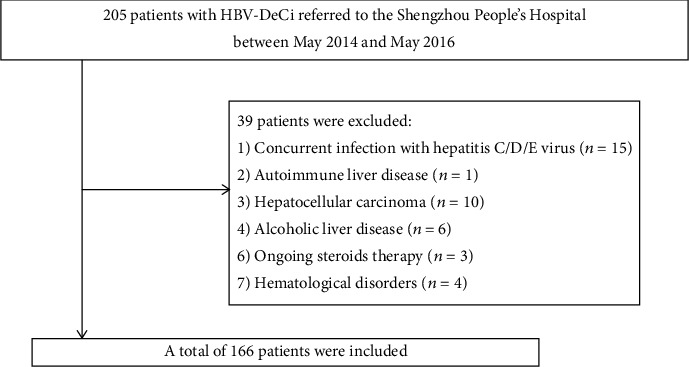
Flow chart of the enrolled participants.

**Figure 2:**
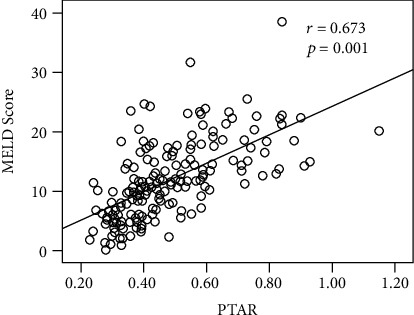
Scatterplot illustrating the correlation between MELD score and PTAR (*r* = 0.673; *P* = 0.001).

After application of the exclusion criteria, 166 HBV-DeCi patients were recruited for the study (Figure 1). The main causes of admission were ascites in 125 (75.3%), gastrointestinal bleeding in 38 (22.9%), hepatorenal syndrome in 17 (10.2%), and hepatic encephalopathy in 4 (2.4%). The median of PTAR was 0.44, and the interquartile range was 0.36 to 0.58 in the patients at admission. As shown in Figure 2, PTAR was positively correlated with MELD score (*r* = 0.673; *P* = 0.001).

At day 30, 27 (16.3%) patients had died. For analysis, the patients were divided into nonsurvivors and survivors according to their 30-day survival. Compared with the nonsurvivors, the survivors had markedly lower creatinine, blood urea nitrogen, bilirubin, INR, MELD score, PTAR, alanine aminotransferase, and aspartate aminotransferase, but significantly higher albumin (all *P* < 0.05) (Table 1).

### 3.2. Factors Associated with Mortality

**Figure 3:**
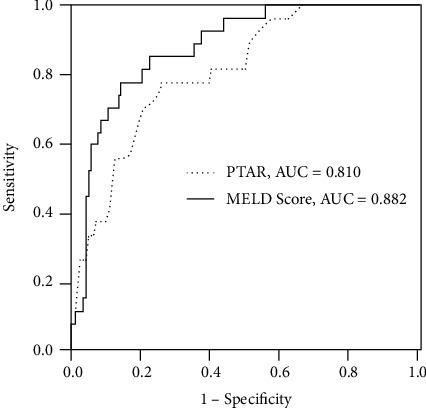
ROC curves showing the relative prognostic performances of MELD score and PTAR for prediction of mortality in patients with HBV-associated decompensated cirrhosis.

As shown in Table 2, MELD score, INR, PTAR, and albumin were associated with mortality according to the univariate analyses. Multivariate analysis identified MELD score and PTAR as independent risk factors for mortality. Subsequently, ROC curve analyses were performed to evaluate the abilities of PTAR and MELD score to predict mortality in HBV-DeCi patients (Figure 3). The cutoff values were 17.21 for MELD score (sensitivity: 77.8%; specificity: 86.3%; positive predictive value: 52.6%; and negative predictive value: 95.2%) and 0.54 for PTAR (sensitivity: 77.8%; specificity: 74.8%; positive predictive value: 37.6%; and negative predictive value: 94.5%). For prediction of mortality, the AUC of PTAR did not differ significantly from the AUC of MELD score (0.810 vs. 0.882; *Z* = 1.534; *P* = 0.125).

### 3.3. Comparisons of Clinical and Laboratory Findings between Patients with PTAR > 0.54 and ≤0.54

The patients were divided into two groups according to the PTAR cutoff value (PTAR ≤ 0.54, *n* = 110 vs. PTAR > 0.54, *n* = 56). Patients with PTAR > 0.54 had higher mortality than those with PTAR ≤ 0.54. Significant differences in total protein, albumin, MELD score, aspartate aminotransferase, total bilirubin, and INR were also noted between patients with PTAR > 0.54 and ≤0.54 (Table 3).

## 4. Discussion

In the present study, the novel prognostic factor PTAR was used to predict poor outcomes in HBV-DeCi patients. We demonstrated that high PTAR was associated with increased mortality. Multivariate analysis identified PTAR as a surrogate independent risk factor for adverse outcomes in these patients.

Currently, several models have been established to stratify the disease severity and assess the prognosis of HBV-associated liver diseases. The most widely used are the Child–Pugh score and the MELD score. However, these scores have some drawbacks. For example, the Child–Pugh score contains five parameters (total bilirubin, albumin, INR, ascites, and hepatic encephalopathy), but the subjective natures for the assessments of ascites and encephalopathy have the propensity to reduce accuracy [9]. The MELD score incorporates three laboratory variables (total bilirubin, INR, and creatinine) and is widely used to evaluate liver function in patients with liver disease and determine the priority of patients for liver transplantation [8]. However, approximately 15%–20% of candidates for liver transplantation are not well served by the MELD score, because the model does not include some important factors (bleeding, ascites, and bacterial infection) that can affect the prognosis [10]. PTAR was initially developed to assess the liver functional reserve in patients with HCC after liver resection [4]. The present study shows that survivors had lower PTAR than nonsurvivors. Furthermore, multivariate analysis indicated that PTAR was a promising predictor of 30-day adverse outcomes, with slightly lower predictive power than the MELD score. Because evaluation of PTAR involves only two common laboratory parameters, it is more easily available and more inexpensive than the MELD score. Of note, previous studies identified some noninvasive models associated with mortality in patients with cirrhosis, including albumin-bilirubin score [11] and C-reactive protein to albumin ratio [12]. Our study complements these studies and demonstrates that PTAR can also be used to predict prognosis in HBV-DeCi patients.

It is well known that systemic inflammation plays a pivotal role in the pathogenesis of HBV infection. Several studies reported that inflammation is relatively common in patients with advanced cirrhosis and linked to poor outcomes [13, 14]. Serum albumin is considered to be associated with various inflammatory responses, with low levels in acute inflammation and an inverse association with magnitude of systemic inflammatory response. We found that serum albumin was markedly lower in nonsurvivors compared with survivors. Therefore, low serum albumin may partly reflect an inflammatory state in HBV-DeCi patients. Meanwhile, albumin is produced by the liver and can act as an index that reflects nutritional status. Low albumin was demonstrated to be a common complication in cirrhotic patients that can lead to ascites or edema and account for their increased mortality [15–17]. In the present study, 125 (75.3%) patients were hospitalized for ascites. In HBV-DeCi patients, hypoalbuminemia suggests malnutrition associated with a decreased hepatic functional reserve caused by chronic liver disease. In addition, liver function impairment is generally associated with adverse alterations in the coagulation and anticoagulation systems. INR also reflects the liver synthetic function, and high INR was identified as a useful predictor for not only increased risk of bleeding but also increased mortality [18, 19]. Furthermore, among the factors comprising the MELD score, INR was reported to have the greatest impact on the score [20]. In the present study, INR was significantly higher in the nonsurvivors than in the survivors. These findings may reflect the hepatic synthetic function, which was worse in the nonsurvivors than in the survivors. Therefore, we speculate that liver cell impairment may lead to reduced production of coagulation factors in the liver as a possible mechanism mediating the increase in INR. In the present study, although INR and albumin were identified as risk factors for mortality by univariate analyses, neither were identified as independent predictors for mortality by multivariate analysis. This difference may have arisen because PTAR is a ratio and is thus more stable than its individual parameters, which may be altered by several factors such as hydration or blood specimen handling. This study also demonstrates a positive correlation between PTAR and MELD score and an association between high PTAR and high mortality, suggesting that high PTAR may be a predictive factor for liver injury severity and progression in HBV-DeCi patients. We note that high PTAR is caused by both increased INR and decreased albumin in this study. Thus, the combination of albumin and INR may specifically reflect the liver function and inflammation and may be useful to predict the prognosis of patients with HBV-DeCi.

Two major limitations of the study are its retrospective nature and small sample size. Another limitation is the lack of dynamic observation of PTAR; thus, it remains unclear whether PTAR became elevated in a stepwise manner as the patient condition deteriorated.

## 5. Conclusions

In summary, PTAR is a useful adjunctive marker for prognosis in HBV-DeCi patients. It is easily calculated and could be used for early identification of patients with high risk of mortality. The present findings will assist clinicians in early identification of severe disease and subsequent prevention and management of this condition. However, further studies are needed to evaluate and verify its applicability.

## Figures and Tables

**Table 1:** Comparisons of demographic and laboratory features between survivors and nonsurvivors in HBV-DeCi patients.

	All patients (*n* = 166)	Survivors (*n* = 139)	Nonsurvivors (*n* = 27)	*P*
Sex (female/male)	33/133	27/112	6/21	0.944
Age (years)	53.0 (46.0-62.0)	53.0 (47.0-62.0)	54.0 (46.0-62.5)	0.920
Total protein (4.40-7.60 g/dL)	6.16 (5.66-6.73)	6.18 (5.76-6.77)	5.94 (2.29-6.53)	0.086
Albumin (2.80-4.40 g/dL)	3.12 (27.2-3.48)	3.17 (2.82-3.53)	2.89 (2.49-3.13)	0.012
Alanine aminotransferase (5-35 U/L)	30.0 (17.0-48.0)	29.0 (16.3-47.8)	39.0 (28.3-61.3)	0.018
Aspartate aminotransferase (8-40 U/L)	46.0 (28.0-73.0)	41.0 (27.0-70.0)	57.0 (41.8-111.8)	0.005
Serum creatinine (15-77 *μ*mol/L)	73.0 (60.0-85.0)	72.0 (59.3-87.8)	84.0 (62.3-118.0)	0.036
Total bilirubin (0.0-21.0 *μ*mol/L)	41.0 (18.0-103.0)	33.0 (16.3-81.3)	131.0 (74.3-274.8)	<0.001
Blood urea nitrogen (1.4-6.8 mmol/L)	5.7 (4.3-7.5)	5.5 (4.2-7.4)	7.1 (5.2-8.9)	0.038
INR (0.80-1.20)	1.34 (1.18-1.59)	1.30 (1.16-1.52)	1.67 (1.46-2.10)	<0.001
Hemoglobin (113-151 g/L)	104.0 (86.0-121.0)	105.5 (85.0-121.0)	101.0 (93.0-112.5)	0.576
Platelet (101-320 10^9^/L)	66.0 (43.0-112.0)	70.0 (43.0-116.5)	63.0 (49.8-82.5)	0.382
PTAR	0.44 (0.36-0.58)	0.43 (0.35-0.54)	0.62 (0.55-0.82)	<0.001
MELD score	11.41 (6.77-17.06)	10.28 (5.93-14.33)	20.33 (17.27-22.67)	<0.001

Data are expressed as number or median (interquartile range). Abbreviations: HBV-DeCi = hepatitis B virus-associated decompensated cirrhosis; INR = international normalized ratio; PTAR = prothrombin time-international normalized ratio to albumin ratio; MELD = Model for End-stage Liver Disease.

**Table 2:** Logistic regression analysis to identify risk factors associated with mortality in patients with HBV-DeCi.

	Univariate			Multivariate		
	Odds ratio	95% CI	*P*	Odds ratio	95% CI	*P*
Albumin (g/dL)	0.908	0.840-0.981	0.015			
MELD score	1.334	1.198-1.486	<0.001	1.286	1.148-1.441	<0.001
INR	19.390	5.520-68.110	<0.001			
Aspartate aminotransferase (U/L)	1.001	0.994-1.009	0.758			
Blood urea nitrogen (mmol/L)	1.002	0.975-1.029	0.898			
PTAR	860.266	55.889-13241.515	<0.001	42.741	1.509-1210.748	0.027

Abbreviations: HBV-DeCi = hepatitis B virus-associated decompensated cirrhosis; CI = confidence interval; MELD = Model for End-stage Liver Disease; INR = international normalized ratio; PTAR = prothrombin time-international normalized ratio to albumin ratio.

**Table 3:** Characteristics of HBV-DeCi patients with PTAR ≤ 0.54 and >0.54.

	Low group (PTAR ≤ 0.54; *n* = 110)	High group (*PTAR* > 0.54; *n* = 56)	*P*
Sex (female/male)	20/90	13/43	0.574
Age (years)	55.0 (47.0-63.0)	51.0 (45.5-58.5)	0.077
Total protein (g/dL)	6.27 (5.87-6.80)	5.80 (5.63-6.45)	0.001
Albumin (g/dL)	3.30 (3.03-3.58)	2.60 (2.31-2.93)	<0.001
Alanine aminotransferase (U/L)	28.5 (16.0-43.0)	37.5 (22.0-62.5)	0.068
Aspartate aminotransferase (U/L)	36.5 (25.5-65.0)	55.0 (39.3-89.0)	0.002
Total bilirubin (*μ*mol/L)	25.5 (15.0-51.0)	96.0 (53.0-197.0)	<0.001
Blood urea nitrogen (mmol/L)	5.50 (4.18-7.60)	5.80 (4.30-7.40)	0.493
INR	1.45 (1.13-1.37)	1.68 (1.54-1.92)	<0.001
Serum creatinine (*μ*mol/L)	73.5 (60.0-84.0)	72.5 (60.5-92.0)	0.744
Platelet (×10^9^/L)	73.0 (46.0-120.0)	63.0 (35.5-88.5)	0.051
MELD score	8.50 (5.36-11.95)	17.45 (13.39-21.20)	<0.001
Hemoglobin (g/L)	106.5 (86.0-122.3)	100.5 (88.0-115.0)	0.323
30-day mortality (yes/no)	6/104	21/35	<0.001

Data are expressed as number or median (interquartile range). Abbreviations: HBV-DeCi = hepatitis B virus-associated decompensated cirrhosis; PTAR = prothrombin time-international normalized ratio to albumin ratio; INR = international normalized ratio; MELD = Model for End-Stage Liver Disease.

## Data Availability

The data are available upon reasonable request.

## Abbreviations

AUCs: Areas under the curve
CI: Confidence interval
DeCi: Decompensated cirrhosis
HBV: Hepatitis B virus
HCC: Hepatocellular carcinoma
INR: International normalized ratio
MELD score: Model for End-Stage Liver Disease score
PTAR: Prothrombin time-international normalized ratio to albumin ratio
ROC: Receiver operating characteristic
